# Reports on the Hospitals of the United Kingdom

**Published:** 1909-12-25

**Authors:** Henry Burdett


### REPORTS
ON THE
### Hospitals of the United Kingdom.

By SIR HENRY BURDETT, K.C.B., K.C.V.O.

### XVI.—COVENTRY AND WARWICKSHIRE HOSPITAL.

Coventry is known as the city of spires, and it presents a most attractive appearance. Its hospital likewise presents some striking contrasts, due to the existence of a number of old wards, which badly need reconstruction, and a number of new and up-to-date buildings, some of which may be regarded as good types of a modern hospital unit.

Thus, on entering the wards, we were seized by a feeling of depression, for they contained more beds than they ought to, light and air were shut out from the end of the ward, and the whole was disfigured by the presence of some fixed wooden partitions which were useless and hygienically dangerous. On calling attention to this matter by an entry in the visitors' book, the House Committee promptly ordered their removal, an action which must have materially improved the hygienic condition of the wards.

The overcrowding is a matter the committee cannot well prevent, for it is forced upon them by the great pressure of work, and they cannot improve matters until additional funds for the extension fund are forthcoming. The floors and fittings, which latter are somewhat meagre, should be renewed, teak being used as soon as possible. Baths, etc., are good. The kitchens, casualty, and out-patient departments are quite out of date. We were glad to see that the latter will shortly be transferred to new buildings, the cost of which is being defrayed by the generosity of the workpeople of Coventry. The kitchens have yet to be dealt with, and they might be provided for in the top of a new ward block, which is called for by the pressure on the beds and the overcrowded wards.

### The New Additions.

Leaving the old buildings, we went across to inspect the new mortuary and the new laundry. Both units are well planned and well carried out. It may be said of them with justice that they contain everything which these departments of a modern hospital should do. Not only so, but they are so planned, especially the laundry, that perfection has nearly, if not quite, been attained. The only minor omission in the laundry that we noticed was want of ventilation in the dry-airing chamber. The mortuary is simple, reverent, and good. It is one of the best of modern mortuaries.

The new Nurses' Home is an attractive building, pleasantly situated and well placed. Each nurse has a separate bedroom comfortably furnished, and the sisters have no difference made in their bedrooms from those of the nurses. The sisters have, however, their own sitting-room, which is the most comfortable and attractive room of the kind we have seen in the provinces. The whole house owes its origin to the generosity of a private patient, the late Colonel Adams, who gave £800 towards the building fund as a mark of his appreciation of the good nursing extended to him by the nursing staff, and as a mark of sympathy with the nurses, who then had bad accommodation. Had he lived, Colonel Adams would have rejoiced over this new home for nurses, and we hope his example and spirit may find many imitators.

We welcomed the new Nurses' Home, because the devotion of Miss Barter, the matron, and her staff of sisters and nurses entitled them to this mark of appreciation. For a modern home the bath accommodation seems a little short.

We are glad to note that the hospital has an excellent chief in the person of Mr. Alfred Herbert, chairman, who is entitled to be proud of the new buildings and improvements which have been added to this hospital during his term of office. It was cheering indeed to find in them such splendid evidence of the right spirit, and we have little doubt that the same spirit will speedily remedy the defects which we have pointed out, and result in the erection of a new pavilion to remedy the overcrowding and provide the necessary kitchen facilities.

Unfortunately Mr. J. A. Rudd, the Secretary and House Governor, was not in the hospital when we inspected it, or we should have liked to inquire of him why it is that in a business city like Coventry the published statements of the accounts are prepared under, but the books of its hospital are not kept upon the Uniform System.

### The Financial Position.

The overcrowding to which we have referred and the condition generally of the older buildings must make this hospital at present a very depressing place for the staff to work in. It is most disheartening to know what ought to be and to find that, try as one will, the appliances and means at one's disposal render it impossible to keep the wards in a state of smart efficiency and to make everything look as it ought to look in a modern hospital. Coventry of comparatively late years has become a centre of active industry, the working men have nobly come foward and supported the hospital, and we hope that other classes of the community will do their share by promptly subscribing sufficient money to enable Mr. Alfred Herbert to go forward and complete the entire reconstruction of this most useful and necessary hospital. The financial position of the hospital at October 31 last was not satisfactory. The accounts show a deficiency of £250 for the year, whilst the Extension Fund shows that £15,000 more is needed to defray the cost of the extension scheme now being carried out.

---

**The Brownlow Hill Workhouse Infirmary, Liverpool,** will be reported on next week.

